# Prospects for Plant-Based Meat: Current Standing, Consumer Perceptions, and Shifting Trends

**DOI:** 10.3390/foods11233770

**Published:** 2022-11-23

**Authors:** Bushra Safdar, Haochun Zhou, He Li, Jinnuo Cao, Tianyu Zhang, Zhiwei Ying, Xinqi Liu

**Affiliations:** 1 National Soybean Processing Industry Technology Innovation Center, Beijing Technology and Business University (BTBU), Beijing 100048, China; 2 Plant Meat (Hangzhou) Health Technology Limited Company, Hangzhou 311121, China

**Keywords:** plant-based meat, consumer perceptions, dietary shifts

## Abstract

Dietary habits have a substantial influence on both planet and individual health. High intake of animal products has significant negative effects on the environment and on human health; hence, a reduction in meat consumption is necessary. The transition towards plant-based meat (PBM) is one of the potential solutions for environmental and health issues. To achieve this goal, it is important to understand the dietary habits and demands of consumers. This review was designed with a focus on PBM alternatives, dietary shifts during the COVID-19 pandemic, the drivers of consumers’ perceptions in various countries, and the measures that can promote the shift towards PBM. The PBM market is predicted to grow with rising awareness, familiarity, and knowledge in the coming years. Companies must focus on the categories of anticipated benefits to aid consumers in making the switch to a diet higher in PBM alternatives if they want to win over the target market.

## 1. Introduction

To ensure future food sustainability and the safety of the environment, several problems must be taken into account, such as climate change, the increasing global population, significant food loss, the risk of new diseases, and pandemic outbreaks [1]. Innovative approaches are required to fully utilize raw resources to minimize the environmental impact of each meal and help the food sector become more sustainable [2]. What we choose to eat has an impact on our health as well as on the future of the planet [3]. A sustainable food system that moves the world’s population toward more plant-based meals and away from animal-based foods is required, since the way we eat today threatens the world through chronic diseases and harm to the climate, ecosystems, and water supplies [4]. Several international food-based dietary guidelines advise consumers to reduce meat consumption because of health concerns [5]. A growing number of people are becoming vegetarian and strive to consume a plant-based diet or reduce their animal-based food consumption [6]. Diets that are plant-based, healthy, and sustainable are frequently preferred by consumers who are concerned about their health and are highly associated with being nutritious and natural. However, this insight adversely impacts taste perception, as it is believed that healthy food is usually not delicious [7]. PBM contains only vegetal ingredients and endeavors to recreate the sensory traits of meat [8]. The most used plant proteins in meat alternatives are soy and pea proteins, which give fibrous texture. Some other plant proteins, such as wheat gluten, potato, mung bean, and rice proteins, are used to improve textural, nutritional, and structural characteristics [9,10]. Pulses are a good source of plant-based proteins and staple foods in various cultures [2]. Several plant-based meat companies are using pulse-based proteins in their products. Fibrous textures and structural arrangements in PBMs are obtained by spinning, extrusion, and steam texturization techniques [11]. Three-dimensional technology is an emerging technique in PBM production, and companies such as Refined meat and Nova foods are using this technique to produce PBM steaks. PBM production techniques and ingredients and their functionalities have been discussed and reviewed in several recent studies [11,12,13,14,15,16,17,18]. PBM products can be appealing to both omnivores and vegetarians as nutritious replacements for meat and provide a sustainable way to obtain the protein they need [19,20]. The PBM market has experienced considerable growth, aiming to substitute animal meat in people’s diets through PBM’s high protein content [21,22]. PBMs are targeted at flexitarians and vegetarians who want to eliminate or minimize their consumption of meat but still crave the flavor, mouthfeel, and texture satiation offered by animal-meat-based foods [23]. Numerous consumers do not consider PBM products to be true substitutes for meat [24]. It might be extremely challenging for consumers to alter their eating patterns, as meat has been central to many people’s meals since the beginning of human civilization and even before [4,25], and they would prefer to stick to their regular diets which they are familiar with and enjoy [8]. However, the transition towards PBM is of vital importance to reduce the environmental and health concerns related to meat consumption without compromising nutritional needs [26]. Currently, a variety of PBM products and brands have entered the market, such as meat cuts, burger patties, sausages, steaks, nuggets, and others. They are promoted as healthy and environmentally friendly options while mimicking the taste and the feeling of eating animal-meat-based products [27].

Although it has been a long time since PBM was launched, no reduction in meat consumption has been observed, and the shift toward PBM is too small and slow compared with the goals to reduce animal meat intake or replace it with PBM products [28]. Although PBM is recognized as a healthier alternative to animal meat, still it is discouraged by some governmental organizations and people worldwide [5,29]. Reasons for discouragement are negative perceptions about technologies, the use of artificial preservatives/additives, its being unnatural, and its production involving a high degree of processing [10,30]. Price is one of the important factors that influences the buying intentions of consumers. The retail price of PBM products is therefore a critical issue for their success and acceptability [22,31]. Previous research on the nutritional quality and production of PBM products has shown that they contain natural plant-based ingredients and are made using safe technologies. However, the question still stands: do PBM products contain artificial flavors and ingredients, or are they healthier and more natural than animal meat [31,32]? So far, encouraging people to replace animal meat products completely or partially in their diet with PBM is challenging because of a lack of cooking knowledge and skills and because of enjoyment, taste, habit, and the belief that meat is necessary for a balanced and healthy diet [24]. Therefore, increasing awareness and educating the public is necessary to modify the dietary habits of people. As people become more familiar with PBM products, the situation may change. Overall, the success of the PBM market is determined by how well PBM is received by consumers around the world [33,34]. It is necessary to understand individual preferences with respect to meat alternatives in order to reduce animal meat demand and promote the shift toward PBM consumption [35], since protein transition (from higher meat consumption to its reduction or substitution through PBM) is highly dependent on consumer behavior [36].

In the above framework, a literature review was carried out to understand consumer behavior and preferences with respect to PBM products in different countries in recent years. Published studies from 2020 to 2022 addressing the present standing and consumers’ perceptions of plant-based meat were included in order to give updated information that has not been reported extensively. Considering this aim, we further deliberated on how to integrate PBM with current values and introduce it into the cooking practices of people. This review is structured in three parts. The introductory section presents the driving forces, importance, and current progress of PBM and the impact of COVID-19 on dietary shifts. The second section provides an overview of consumer attitudes and the drivers of consumer behavior. Based on the second part, the final section discusses the responses to these attitudes and considers whether and how they are evolving to show how PBM alternatives might gain in popularity over time.

### The Present Standing of PBM and Shifting Trends

**Figure 1:**
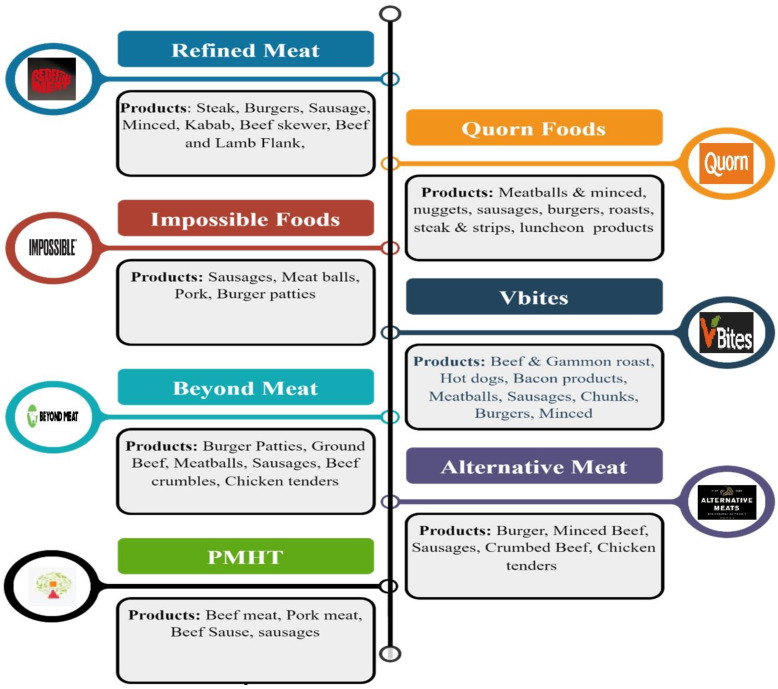
Main products of some plant-based companies of different countries.

The growth of the PBM market is expected to accelerate in coming years, and this emerging market seems to be well-positioned for potential development and innovation [9]. Despite becoming more popular, the market for plant-based meat substitutes still has a small market share compared to animal meat [29]. Negative perceptions, animal meat attachments, reduced sensory quality, dietary habits, and the lack of familiarity with these products may cause lower acceptability [37,38,39,40,41]. Several studies have reported that higher intake of animal meat has been associated with the pathogenesis of non-infectious diseases [42,43,44]. On the other hand, growing research suggests that diets high in plant-based foods improve health and induce changes in gut microbiota composition [30,31,32,45]. Therefore, people who are concerned about their health are becoming vegetarians or looking for products that do not contain animal products [18]. People can reduce their intake of or substitute animal meat with meat alternatives without compromising the feel and taste they obtain from it [46]. The rise in globalization has significantly changed people’s understanding of the food they need. Farmers used to provide food for adjacent cities before globalization, and there was limited choice and availability of food [15,47]. People believe that nutritious and healthy foods are to be directly obtained from natural sources without any processing [48]. Thus, consumers must have a fundamental understanding of food production, preservation, and preparation. Due to the growth of the food economy, demand-driven rather than supply-driven food systems have arisen, and in this shift consumer preferences play a significant role [49]. The development of products that are easy to incorporate into current eating patterns is necessary in order to move toward plant protein consumption, which would lead to a decline in the consumption of meat [12]. Subsequently, PBM products that closely resemble animal meat products have been developed [50]. Marodin [51] stated that it is very challenging to get people to adopt new foods, particularly when these new products are being used to replace ones that consumers like and which form major parts of their meals, such as meat. It has been observed that the meat supply chain was disrupted during the COVID-19 pandemic and that PBM companies seized the opportunity to attract new consumers with more competitive marketing strategies [52]. As the PBM industry is growing and new products and brands enter the market, companies may use advanced techniques to improve the nutritional values of PBM foods to make them comparable to those of meat products [1]. PBM is now accessible in numerous supermarkets all around the world, and it is also becoming more common in well-known restaurant chains [5]. New options for every kind of animal protein alternative are available in grocery stores [9,34]. In this way, the decision to eat more plant-based meals and reduce meat consumption has become easier with so many options to choose from [15]. A list of some pioneers in the PBM industry and their major products is presented in Figure 1; the most common products for all companies are steaks, sausages, patties, and minced meat. The competition among companies drives them to consider ways to improve existing products and develop new products that are associated with various consumer-related and technological challenges [52,53]. When PBM products enter the markets, it is important to consider the properties, values, composition, and usage patterns of meat products, as well as the local customs and culinary preferences of specific areas [29]. Regular dietary patterns including meat or plant sources are differentiated by behaviors and practices and have significant social and cultural impacts [36].

The market share of meat alternatives is still small, and further research is needed to determine how to increase the consumer acceptability of PBM alternatives. Recently, numerous studies have been conducted regarding the improvement and nutritional/health benefits of PBM. Some of these studies are summarized in Table 1. In a survey, Carlsson et al. [28] found that 30–40% of people had a lower willingness to choose a veggie burger even when given one for free because they were unaware of the flavor, taste, texture, appearance, and cost of PBM. Negative perceptions are more frequent in people who have not tried PBM products or who like animal meat and do not want to reduce their meat intake at any cost [54,55]. Elzerman et al. [56] suggested that not everyone needs to go vegetarian but that reducing intake of meat a few days a week could help transition towards PBM and possibly lessen health-related issues associated with higher meat consumption.

## 2. Dietary Shifts during COVID-19

**Figure 2:**
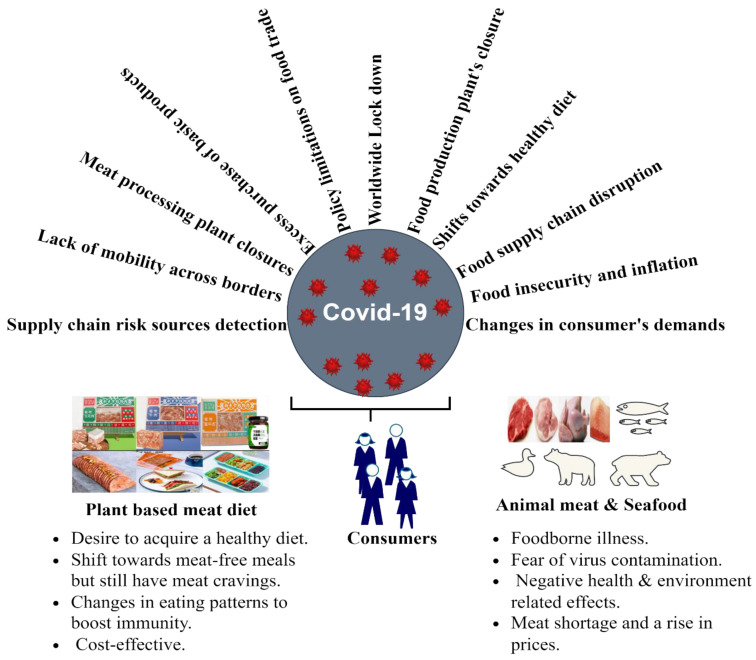
Changes in the food supply chain and the dietary behavior of people that occurred during the COVID-19 pandemic.

Food security is based on having access to affordable, safe, and healthy food. Nearly all economic sectors, including agriculture, have been affected by the COVID-19 pandemic, and the word “important” has been frequently used regarding the food industry [69]. COVID-19 resulted in the closure of food manufacturing facilities, restricted food trade policies, and subsequent changes in consumer demand, and thus the food supply chain has been under financial pressure to produce products necessary for daily life [70]. The pandemic served as a reminder of our dependance on a global food value chain and our susceptibility to interruptions in this important sector [71]. In countries that depend on seasonal migrant workers in the agri-food sector, a sudden lack of mobility across borders and within countries led to labor shortages, which in turn had an impact on the availability, supply, and cost of food globally [72]. On the other hand, the great infection rate among employees at slaughterhouses and meat processing facilities was of particular concern. During the subsequent quarantine and lockdown periods worldwide, disruption to the meat supply chain was noticed and almost everyone faced one of the major issues, i.e., food insecurity. Consumers suffered from meat shortages, price fluctuations, and meat safety issues because of the closure of meat handling plants. Taking into account that meat shortages may cause prices to increase, consumer demand was further reduced [52,58]. There was an imbalance in all food supply chains, and the main drivers of dietary shifts were disease prevention and better health status [2,60,73]. As a result, the pandemic put the world’s population at risk of zoonotic infections spreading to humans through the consumption of food products derived from animals [74]. The influence of the pandemic on the food chain and on dietary shifts in consumers is represented in Figure 2.

When food is served in a restaurant, most people do not give much attention to how it was prepared or the ingredients that were utilized. However, concerns about food safety during the worldwide epidemic have brought attention to the restaurants and staff responsible for providing a safe and reliable food supply [75]. Since the start of the epidemic, there was a decline in meat sales, largely ascribed to restaurant closures. When dining out, meat is frequently preferred over vegetarian options—a choice that was temporarily unavailable for many people—and meat became more expensive [2,63]. As a result, there was a remarkable shift towards plant-based meals and the demand for PBMs increased. During the epidemic, many people shifted to a plant-based diet because they thought it might boost immunity or provide other health benefits in addition to being more economical [74]. Loh et al. [52] conducted a Google Trends data study and observed that searches for “vegan/veganism” were at an all-time high, and sales of plant-based foods rose during the 2020 pandemic, while those of animal meat and seafood declined due to distrust of meat and seafood. Tonsor et al. [35] surveyed the effect of the pandemic and lockdown on meat consumption in the US and found diverse influences across households on hoarding behavior and financial confidence and shifts in consumers’ financial conditions throughout the pandemic. Hence, the peak month for purchasing plant-based food was March 2020, when sales increased by up to 90% in contrast with March 2019. The current pandemic presented an opportunity to reduce meat consumption, which could have long-term positive effects on both health and the environment if the COVID-19-induced rapid dietary modifications are maintained [75]. Additionally, the COVID-19 pandemic also revealed considerable adaptability within the food system and highlighted the nature of the supply chain, which is marked by high levels of cooperation and efficient communication, the minimization of transaction costs, and a tendency to be more flexible in facing such challenges. Thus, after the COVID-19 epidemic, the food industry should be better prepared to withstand severe disruptions, having identified key sources of supply-chain risk and taking precautions to control these risks [57,58,59]. It is still unclear whether people will continue to eat less meat once the current pandemic has passed or whether previous dietary patterns will resume as life returns to normal [76]. If the dietary changes that were observed during the outbreak are not incorporated into consumers’ daily routines, they might not be maintained [2]. The focus should be on preventing, or at least limiting, the usage of wild animals, primarily as a source of food, in order to control future zoonotic diseases. The development and implementation of practical, data-driven solutions should be undertaken in collaboration with international multidisciplinary health authorities and specialists [74]. In order to promote a healthy diet, well-known governmental organizations, social media, the guidance of health professionals, and the provision of incentives to restaurants, schools, and hospitals to offer plant-based food options could play a significant role [52].

## 3. Opinions about and Predilections of Consumers for PBM around the World

Consumer behavior and preferences are a dynamic field of study that enables marketers to understand and predict the choices, purchasing behaviors, and preferences of consumers. For the success of a new food product, consumer acceptability and perceptions are crucial since they determine whether it will be accepted or rejected. Therefore, negative opinions are associated with the failure of an innovative product [44,64,77]. The demand for meat and meat products will continue to rise as the world’s population grows. In order to lessen the burden of meat production from an ethical, environmental, and dietary perspective, switching to PBM could be a possible alternative [78]. Although there is still debate over PBM acceptance, it is crucial to understand customer demands to improve the quality of PBM and deliver similar textures and flavors that are comparable to those of animal meat products [24,79]. Country and region are relevant to consumers’ perceptions and motivations, due to the differences in cultures, attitudes, traditions, environments, and behaviors across the world [66,67]. Up till now, various surveys have been conducted about consumer acceptance of PBM in various countries, and, due to cross-cultural differences, varied responses have been recorded. Table 2 details the previous studies on consumer behavior and preferences in different countries which may contribute to understanding the diversity of responses to PBM around the world. The recent growth in the PBM sector is associated with the interest in healthier dietary patterns. In addition, there is a trend toward reducing meat consumption or switching to a flexitarian diet in order to increase nutrient density while still eating meat occasionally [80,81].

Some people who want to reduce meat intake consider PBM alternatives that are similar to animal meat. Therefore, to increase PBM consumption, there is a need to formulate PBM products that mimic all the characteristics of meat products and offer substantial value for money [40,54]. Complete nutritional information on the pack—especially nutrient contents in comparison with those of meat—is essential to avoid PBM from being rejected by health-conscious consumers [94]. Products with functional ingredients are preferred by consumers because, in their view, functional ingredients are linked with health benefits [14,47]. PBM acceptance is more challenging for people who habitually eat animal meat [43]. According to a survey by Michel et al. [35], omnivores preferred meat, but vegans considered that PBM was better with respect to flavor, texture, price, being easy to cook, fat and protein contents, and environmental friendliness. Flexitarians believed that meat was better in terms of taste, texture, and cost, while PBM alternatives were thought to be superior in relation to fat content and environmental friendliness. Vatanparast et al. [33] suggested that the partial substitution of animal meat with PBM (hybrid meat) may gain more acceptance and might boost dietary intake of several nutrients while limiting cholesterol intake, though it would have a negligible impact on sodium and saturated fat intake. The demand for novel and sustainable proteins will rise as more people become aware of health and environmental issues. However, some people (who habitually eat meat daily) are resistant to reducing meat intake because of taste, sensory attributes, habit, adherence to social norms, and considering meat to be important for good health [31,33,66,71]. Hwang et al. [6] found that people with high food curiosity (the strongest motivating factors that might make people try new food) are more likely to buy PBM; however, it would take a tough and long effort to make them permanent consumers (especially those who love eating meat daily). Moreover, consumers anticipate that PBM should be more affordable, higher in protein and vitamins, and lower in calories when compared to meat [36,72].

## 4. Barriers to Switching Diet towards PBM

The barriers concerning the consumption of PBM vary for different consumer groups; thus, an intervention that is effective for one group of consumers may not be successful for other groups [25]. The main barriers to PBM are consumer beliefs, familiarity, or previous experience with PBM products and environmental, health-related, ethical, nutritional, and sensory aspects [5,36,87,95]. Carlsson et al. [28] reported that lower acceptance of PBM in Asian countries compared to Western countries is due to cultural differences, food habits, availability, and accessibility issues, and, most importantly, awareness. Appearance, texture, and flavor are key aspects influencing the acceptance and consumption of products [23,36,47,49,69]. Various studies have found that price, brand name, gender, food neophobia, education and awareness, nutrition, labeling, environmental concerns, and technological information influence the selection of meat or PBM [6,22,25,42,46,54,55,74,75]. Consumers remain hesitant to try unfamiliar or new food products, resulting in negative preferences [96]. Another barrier to acceptance of PBM is technological distrust, as consumers generally reject advanced technologies, such as genetically modified foods, due to concerns about safety and naturalness [22]. A big hurdle in PBM acceptance is associated with beliefs about sensory attributes, taste, and PBM products being incomplete/unsatisfactory, as people may think that meat products taste better than PBMs, even when all the products in question have the same taste and appearance [7]. The degrees of food neophobia that consumers experience directly affect their meal choices [26]. A variety of cues (culture, hobbies, habits, morals, and qualities) have direct impacts on the performance (positive or negative) of products in the market [44]. People who habitually eat meat dislike PBM. Thus, PBM typically appeals more to people with less meat attachment [82]. Marodin [51] demonstrated that the description of PBM products (e.g., as plant-based, meat alternatives/substitutes, vegan, or veggie) affects consumer product perceptions and related behavioral intentions. According to Li [97], consumers will more readily accept a product of a better-known brand because they believe that reputation, quality, and sales are correlated. Cordelle et al. [79] observed that, even though willing to try new products, consumers’ intentions to spend more money on PBM were found to be low due to lack of knowledge. The more often consumers are exposed to new food products through knowledge, the lower the possibility of rejection [84]. However, familiarity with and understanding of new products tend to increase with education [60,79].

## 5. PBM Pleasure Drivers and Factors Involved in Product Success

### 5.1. Nutritional and Sensory Qualities

Sensory properties have a significant impact on consumers’ purchasing decisions; hence, it is important to improve sensory qualities to ensure product acceptability [95,98]. This is particularly true for products intended to replace meat, as their success is based on sensory qualities [48]. The primary barrier to the acceptance of PBM products is considered to be their perceived sensory attributes in contrast to those of meat [23]. Previously, it has been reported that the success of PBM is dependent on flavor, sensory, and nutritional properties [2,27,38,55,66,79,80,81,82]. Additionally, lack of familiarity and poor sensory appeal have been cited as the main barriers to PBM acceptance [80]. It is important to pay attention to the fact that PBM must have nutritional characteristics equal to or comparable to those of animal-based products. Kurek et al. [18] reported that a product is deemed to be of high quality if it contains more than 30% protein, is low in fat, and has an amino acid score that are similar to a meat protein digestibility-corrected amino acid score. Kaczmarska et al. [38] described that taste as an important factor because, for many consumers, the first experience is decisive. Indeed, if they make the effort to taste a product that they do not know and if they do not like it, they may have a bad view of the entire line of PBM products, which would significantly reduce their possibility of becoming a regular consumer of PBM. Wang et al. [23] described that the flavor profile of the final product is greatly influenced by the protein source. Understanding the processes by which flavors and off-flavors are developed in PBM products will help in forming efficient ways to reduce their off-flavors and improve their preferred flavors. In a survey, Caputo et al. [88] found that product names/labeling and taste are major factors in PBM acceptance. According to Ping et al. [47], the similarity of PBM to meat, particularly in terms of texture, taste, and flavor, is one of the prerequisites that is currently impeding the move from the consumption of meat to PBM substitutes. Estell et al. [20] reported that PBM products should be like meat regarding texture, taste, and color to encourage repeated purchases. According to Guo et al. [57], texture and flavor are the key elements influencing consumer acceptability. In the extrusion process, several factors are involved in the retention or loss of volatile compounds, including raw material composition and moisture content, extrusion conditions (residence time, extruder temperature, compression and pressure, vapor loss during expansion, and diffusivity of the volatiles in the mass). Elzerman et al. [56] reported that PBM products that mimic the sensory characteristics of meat may appeal to consumers who frequently consume meat. However, when sensory characteristics do not meet consumer expectations, even products with health benefits may be rejected by customers [85]. According to Hobbs [70], contrary to person-related beliefs (such as taste and environmental or health concerns), product-related beliefs (including sensory characteristics and product details) are considered when buying a meat substitute which could influence consumer attitudes and the acceptance or rejection of products. de Oliveira Padilha et al. [84] found in a survey that PBM is thought to be less palatable; as the taste of PBM continues to influence dietary choices, these perceptions may act as barriers to the substitution of PBM for animal meat. The use of a variety of additives to generate a meat-like texture, juiciness, tongue feel, and flavor raises questions about sensory qualities, nutrition, clean labeling, and consumer confidence [66,74]. In addition, PBMS should be as clean as possible and made with natural ingredients to fulfil consumer demands with respect to health benefits [30]. Familiarity with food items plays a significant role in reducing uncertainties about products and results in better matches between expectations and sensory attributes [42,80,98].

### 5.2. Consumers’ Attitudes towards the Consumption and the Price of PBM

In daily life, several interconnected situations frequently have an impact on eating habits. The knowledge of foods is one of these elements. People often choose meals that involve less processing and are easier to prepare when they are dining at home, which is a trend in modern living that is seen in many different countries [5,22,85]. Vaskelainen et al. [53] described that consumers want quick- and easy-to-cook food that can be fitted to everybody’s taste and is suitable for all palates. According to Sha and Xiong, [9], fast foods, such as burgers and pizzas, are associated with status-related elements that may affect people’s desire for more conventional selections when trying to set themselves apart from their social classes. Modern foods are seen to be associated with high social status, while more traditional cuisines are thought to be associated with low social status. Anusha Siddiqui et al. [78] reported that beliefs and attitudes related to PBM still need to be influenced, considering all the psychological factors involved in the success of a new product. The launch of new products is also essential to offer more choices to the consumer beyond classic meat products, such as burgers, minced meat, sausages, and nuggets [15,54]. Additionally, the price of PBMSs need to be reduced to make these products attractive to the public [77,99]. As some consumers compare PBM products to meat, the most effective strategy would be to set them below meat. Before reaching this price level, a discount strategy is recommended, as it would allow consumers who find substitutes too expensive to try them, to potentially have a good first experience, and then to buy them again, even though they become more expensive [55,75,86].

### 5.3. Awareness of New Technologies and Novel Foods

Food neophobia is a typical response that manifests when new technologies are used or when food defies consumers’ food/gastronomic cultures [66]. Some people believe that new technologies violate norms and are not ethical, producing moral issues and leading to the failure of food [22,84]. Uncertainties, health and environmental risks, and concerns about new technologies are important issues to be addressed, as they are associated with the acceptance of products. Michel et al. [35] reported concerns about whether those who are motivated to eat more environmentally friendly foods have the knowledge necessary to make the right judgments and whether good intentions could lead to poor choices due to ignorance. However, better information and knowledge may help people choose sustainable foods, and an information interpretation guide may also be required [89]. According to Jung et al. [66], PBM is becoming more popular among consumers as an alternative to animal meat because it is a sustainable protein source and does not raise any moral dilemmas. To meet the expanding market demand, less expensive and energy-intensive processing methods should be considered.

### 5.4. Food Literacy and Cooking Ability

The ability to manage, choose, prepare, and consume healthy foods to fulfill needs and determine intake is known as food literacy. It is a modern idea that gives a framework for comprehending the connection between food related knowledge, skills, and dietary consumption [41,82]. Nutritionists have long been interested about how people go about feeding themselves. They have considered cooking, nutritional knowledge, food involvement, food safety, and food skills [4,32]. The concept of food choice value guides individuals when choosing foods to buy or consume [37]. In daily life, food choices are often habitual and influenced by many interacting factors. To promote the consumption of PBM, food literacy is needed. Additionally, cooking ability is crucial for the adoption of any food ingredient since understanding how to use the ingredients and which recipes they complement can increase their demand and acceptance [39,87]. In addition, when new products enter the market, most consumers do not always know how to use them. Thus, consumers should be provided with recipes or be informed about ways to easily integrate the products into their routines. Since cooking expertise is thought to be learned through practice, a consumer’s level of sensory discrimination may vary depending on their level of culinary ability. Consumers with less or no cooking skills may pay less attention to a food’s intrinsic characteristics than consumers with better skills, which could be a result of a lower level of interest in food [3,41]. According to Hellwig et al. [48], it could be helpful to use recipes from traditional food-cooking cultures while preparing new products. Therefore, considering how food is cooked might help improving flavor and taste. Serving new foods with ingredients that peoples are already familiar with does not always improve their willingness to try them. Cuisine recipes featured in the media also reflect the originality and tradition of products. The use of PBM as a meat substitute in recipes supported by renowned chefs should have included both classic and modern dishes (in Asian cuisine, for instance) [53].

### 5.5. Influence of Distribution Channels and Promotion

A whole new set of relations must be established around a new product when it is brought to the market because it is likely to be unfamiliar to customers, retailers, and legislators [6,34]. Therefore, it is crucial to present the products appropriately so that consumers can locate the products easily in supermarkets and make purchasing decisions [36,74]. It is also necessary to keep making PBM products more noticeable and prominent in supermarkets to reach the maximum number of customers. It would be better if they were situated near the meat so that customers could easily find and compare them with meat [15,85]. It is also recommended to distribute PBMSs in restaurants to encourage consumers to try them and potentially adopt them afterward. The advantage of restaurants is that they can showcase PBMs and give consumers ideas on how to use these products in their regular meals [26,88]. Social media and the internet can increase potential benefits by reducing physical distances, allowing businesses to increase the possibility of expanding their markets, and considering that the development of direct relationships between producers and end users can result in long-term advantages for PBM acceptance [7,53,72]. According to Septianto et al. [100], social media posts with profound colors and vertical and horizontal symmetry obtain higher numbers of likes. To have an impression of the commercial events of a company, promotion is an essential component. Celebrity-endorsement advertising, which is popular in the global market by fusing social culture with marketing, is one of the most effective tactics [97]. Vaskelainen et al. [53] suggested that hype (an increase in public awareness and expectations about innovation potential, such as media hype) can quickly ignite the demand for a sustainable product and create a bandwagon effect that brings many similar niche products into the mainstream, which is necessary to enable transitions toward PBM.

## 6. Effects of PBM on Rural Life and Animal Farming

The worldwide demand for meat has increased by 58% during the last two decades. By 2050, the population of the globe is expected to reach 9.7 billion, according to UN predictions. Thus, it is anticipated that there will be a sharp rise in the demand for animal products worldwide in the coming years due to increasing population and per capita utilization [15,68]. Numerous economies rely heavily on the animal husbandry industry, and the production and consumption of animal products is often rooted in long-standing social and cultural customs [24]. However, the production of meat is becoming more challenging because of problems such as water and land shortages, animal safety, and climate transformation [12]. PBM analogs that resemble traditional meat in taste and texture are thus being developed to meet the growing population’s need for protein. The demand for different crops as source of plant proteins is likely to increase as the market for PBM expands, which will open a range of options for farmers who already cultivate such crops or who might add them to their rotations [96]. However, some people and communities (who depend on animal husbandry for their livelihoods or commercial viability) perceive PBM as a direct threat, though the rise of the PBM industry may not pose a threat to the sustainability of the animal meat sector [5,10]. In general, it is quite unlikely that people would entirely switch from animal meat to PBM. The primary purpose of PBM would be to satisfy expanding protein demands rather than completely replace animal meat products. The fear that traditional livestock farmers/ranchers will inevitably experience financial hardship or lose their means of subsistence is unfounded [81]. Additionally, PBM can also help feed people in poor countries and disaster-prone regions (e.g., flood and earthquake zones) to fulfill their protein needs, where food supply and preservation are difficult. Thus, the capability of PBM to satisfy the demand for protein in poorer or developing countries is strictly subject to affordable price levels.

## 7. Economic Sustainability

The ability of the PBM industry to price its products competitively with respect to conventional meat is one of the keys to success. The higher consumer acceptability of PBM depends on price equality with animal meat, which is related to the capability of the industry to scale up production [31,101]. PBM needs to become more affordable to compete with animal-based meat products, as affordability is associated with purchasing intentions. Price is an important factor that must be taken into consideration to make PBM appealing to consumers and to gain market share [64,73]. In addition to taste and texture, a competitive price is crucial for market penetration, as price is a factor that could either encourage or discourage consumers from buying PBM products [102]. Consumers are often very familiar with meat prices and are therefore price sensitive to PBM. PBM products are still priced at a premium compared to conventional meat [103]. Garnett et al. [102] found in a field experiment that small price changes can raise vegetarian and vegan meal sales and reduce sales of fish and meat, but only for diners with higher prior levels of vegan and vegetarian meal choices. The cost of plant-based proteins was found to be a huge barrier for people looking to include plant-based foods into their diet. 

## 8. Conclusions

Consumer acceptance is the main factor in the success of any food product. Consumer perceptions about PBM vary greatly across the world due to differences in culture, behavior, taste, and food habits. However, many factors are similar, e.g., familiarity with food, sensory and textural preferences, and certain food motives. Focusing on knowledge and practical function as paradigms to predict and describe PBM adoption may result in long-term dietary adjustments. Alternative meat products that are similar to meat in terms of appearance, sensory attributes, and cooking may have higher acceptance rates. Food industries must understand the demands and advantages expected by consumers who desire to lower their meat intake. PBM products must satisfy the sensory preferences and expectations of consumers. In addition to taste and texture, price is an important factor that must be taken into consideration to make PBM appealing to consumers and gain market share. In the future, there should be a focus on improving technology and resources to make PBM affordable and improve the texture, nutritional value, and sensory attributes with low environmental impact.

## Figures and Tables

**Table 1:** An overview of the previous findings (2020–2022) about plant-based meat effectiveness and products.

Subject Matter	Analyzed Aspects	Verdicts	Reference
Addition of wheat gluten in MA	Influence of wheat gluten and high moisture extrusion on the volatile flavor compounds, protein structures, and microstructure of MA	The retention rates of volatile flavor substances were affected by extrusion, wheat gluten, and moisture levels in the raw materials	[57]
Textured-wheat-protein (TWP)-based MA	Impact of the fibrous structure under different moistures during extrusion on the protein digestibility of TWPs	The extrusion moisture of textured wheat protein had a significant effect on the fibrous structure, which affected the digestion of wheat protein in TWPs	[21]
Pea- and microalgae-protein-based MAs	Pea protein and *Haematococcus pluvialis* residue (HPR) were used in MA production employing HRE	The incorporation of HPR improved the appearance of MA. HPR can be used as a raw material for HRE to enhance the physical properties of MA	[58]
Novel TVP containing rice protein isolate	SPI was replaced by rice protein isolate (RPI) in various amounts in TVP production by LME	RPI and SPI combination in TVP improved the nutritional value of MA. RPI has the potential to partly replace SPI in MA production	[11]
Enhancing functional properties of MA	Development of a novel binding system using laccase, proteins, and SBP to improve the binding ability of petties	The presence of laccase and SBP improved the moldability and binding ability of MA patties. It could be an effective binding system for MAs	[14]
Binder system for bacon analogue (BA)	Preparation of SPI-based binding system and factors influencing the ability to assemble the BA	The BA could be adhered by an SPI suspension and gelled by heating or TG addition. Binder strength can be achieved with coagulated or TG-cross-linked SPI gels	[13]
Environmental impact of meat and plant-based burger patties	Soymeal and pumpkin seed flour was processed using HME and LME to obtain TVP to produce plant-based burger patties	Plant-based patties had a lower overall environmental impact than meat-based patties. High-moisture extruded soymeal TVP was found to be the most environmentally feasible option	[59]
Plant-based versus animal-ground beef	Cooking quality and sensory characteristics	GBA provides different eating and quality experiences to GB, varying in color, cooking characteristics, and texture	[60]
Plant-based salami analogue	Formulation of novel animal fat replica using different levels of sal fat and canola oil for plant-based salami	Solid fat addition into the lipid phase of fat particles can improve the appearance of the desired fat marbling in the product, without affecting sensory properties	[50]
Plant-based nuggets (PBNs)	Development of optimal composite of pea and wheat protein to make PBNs using a freeze structuring technique (FST)	FST ensued a fibrous and layered structure of the nuggets, and wheat protein contributed to the texture, which closely resembled meat texture	[61]
3D-printed reduced-fat MA	Reduced-fat MA was produced using biosurfactant variants and printed using an extrusion-based printer	3D printing technology can create MA with anticipated 3D structures and modified textures for improved eating experiences	[62]
Flavor and metabolite profiling of meat, MA, and THPPBF in Australia	Classification of the volatile and non-volatile flavor metabolites produced in grilled meat and comparison with commercially available MA and THPPBF	Greater diversity and a large quantity of volatiles were observed in PBPs compared to grilled meat. A variety of free amino acids, di-tri-tetra-peptides, nucleotides, flavonoids, and other metabolites were detected in all products	[38]
Meat and PBMS in the Norwegian market	Comparison of the macronutrient and salt contents of PBMS with those of meat	Meat and PBMS differed in terms of nutritional composition. PBMS contained lower levels of saturated fat and higher levels of fiber	[63]
Fibrous structure and nutritional profile of MA	Mechanical and physicochemical classification of MA containing blends of pea protein isolate and oat fiber processed with HME	Addition of certain levels of soluble and insoluble dietary fiber resulted in fibrous MAs with diverse mechanical and physicochemical properties with improved nutritional value	[64]
Nutritional quality evaluation of PBMAs in the Swedish market	Nutritive quality assessment of PBMA by NC to RNI, labeling schemes (nutri-score, nutrition claims, and keyhole) and comparison with animal meat	Most PBMA classes are healthful choices compared to animal meat, primarily due to their higher fiber contents and lower saturated fat contents. For salt, various PMBAs are healthy substitutes for processed meat but frequently less healthy choices compared with unprocessed meat references	[65]
Health- and nutrition-related claims made by MA companies	Analysis of nutritional and health claims on the companies’ webpages and front-of-pack labels in the US	Companies are using nutritional marketing to promote their products in relation to meat. There is an emphasis on ingredient and nutrient claims, but processing-method information is neglected	[27]
Nutritional quality of MA products	Nutritional and health claims, ingredient details, and organic and gluten-free declarations were collected from the labels of 269 MA products on the Italian market and compared with animal products	MA products showed a longer list of ingredients than animal meat products. PB steaks, cutlets, and cured meats exhibited some favorable nutritional aspects compared to animal-based products	[55]
Production of SPBMA using ohmic cooking	Influence of ohmic cooking system on SPBMA	Ohmic cooking presented good energy efficiency, textural qualities, and uniform temperature distribution during the cooking process	[66]
Non-linear rheology and heating of meat and MA	Texture maps and dissipation color patterns were used to observe similarities and differences between rheological responses of meat and MA	MA had similar stress and strain values but lower elasticity compared to heated meat. Heating of meat resulted in tougher and more elastic material, while MA showed little effect of heating	[67]
Effect of PBM on consumer gut microbiota	A randomized controlled test to evaluate the variations in the gut microbiota of 20 participants who substituted various meat-based meals per week with PBMAP	The animal meat replacement with PBMAP in flexitarian food may have a positive impact on the gut microbiome	[46]
Protein digestibility of real and PB pork and beef	Evaluation of in vitro protein digestion (gastric phase, intestinal phase, and gastrointestinal digestion)	The digestibility of both products was different and was related to protein secondary structure, disulfide bond formation, and digestion solution viscosity	[30]
Digestibility and gastrointestinal fate of meat and PBM	Gastrointestinal fate was compared using the INFOGEST in vitro digestion model	PBM and animal meat are associated with different digestion mechanisms. There were several differences in gastrointestinal fate due to the presence of dietary fibers, SPI, and oil in PBM	[45]
MA preservation by antimicrobial film (AMF)	AMF was made with cinnamaldehyde/tea polyphenols, polylactic acid, polybutylene adipate, and starch blends, using the extrusion technique	AMF successfully inhibited evaporation and maintained the texture properties, quality, and safety of MA	[68]

MA = Meat analogue, HRE = High-moisture extrusion, TVP = Textured vegetable protein, LME = Low-moisture extrusion, RPI = Rice protein isolate, SBP = Sugar beet pectin, TG = Transglutaminase, GB = Ground beef, GBA = Ground beef alternative, THPPBF = Traditional High-Protein Plant-Based Food, PBMS = Plant-based meat substitute, NC = Nutritional contribution, RNI = Recommended nutrient intake, TSPI = Textured soy protein isolate, PBF = Plant-based food, PBPs = Plant-based products, HME = High-moisture extrusion, SPBMA = Soy-protein-based meat analogue.

**Table 2:** Overview of studies aiming to understand the acceptance and perceptions of plant-based meat in different countries.

Aim of Study	Country	Method	Finding	Reference
Factors influencing PBM and CM buying intentions	Korea	An online survey in two separate sections for CM (*n* = 513) and PBMA (*n* = 504)	Sustainability, food neophobia, food curiosity, unnaturalness, and suspicion of biotechnology influence buying intentions	[6]
Drivers and inhibitors of consumer attitudes and buying intentions towards AMP	China, USA, France, Spain UK, NZ, DR, Netherlands, and Brazil	Online surveys including 3091 participants from nine countries	Food neophobia is the main inhibitor, while nutritional value, environmental impact, healthiness, and sensory attributes are drivers for PBMA acceptance	[19]
Preferences and buying intentions of consumers for PBM and CM vs. conventional meat	Spain	A choice experiment involving 444 participants (recruited via e-mail, using research databases)	In terms of preferred items, there were three types of consumers, i.e., price-sensitive millennials and concerned (target group for PBM industry) and indifferent consumers (high willingness to pay for PBM)	[22]
Consumers’ attitudes towards PBM and CM over two consecutive years	Belgium	Cross-sectional online surveys using CAWI in 2019 (*n* = 1001) and 2020 (*n* = 1000)	Positive attitudes towards PBM increased from 44% (2019) to 51% in 2020. PBM was more appealing to younger consumers	[82]
Consumers’ perceptions of MA and PRF	Switzerland	534 participants evaluated the healthiness, environmental friendliness, and naturalness of 20 PRF and MA products	Most participants did not consider MA products to be healthier or more environmentally friendly because they are meat-free	[49]
Consumers’ attitudes toward PBM products	Turkey	Content analysis of different Turkish food web forums	Negative remarks: Unhealthy, tasteless, unusual, negative in general, and expensive. Positive remarks: Healthy, needed, environmentally and animal-friendly, secure food, sustainable, and tastes good	[83]
Consumers’ attitudes towards CM, FRM, and PBMA	Australia	The attitudes of 1078 participants were determined across six attributes: health, protection, price, eating pleasure, animal safety, and eco-friendly attributes	Beliefs about the price and health of FRM were strong. For all attributes, beliefs relating to PBMA were more positive than those relating to CM	[84]
Consumers’ opinions and associations with traditional meat vs. AM	Germany	Online survey (1039 participants) conducted by a commercial panel provider, RAG (CG)	Traditional meat is linked with positive terms compared to AM. Eating place and companionship influence meat choice	[35]
Opinions of consumers and nutritionists about PP and PBMA products	Australia	Cross-sectional self-administered online survey via social media with 679 responses	Taste was considered vital across both groups. Health was a key determinant of diet type among nutritionists, while ethical reasons were mentioned by consumers	[20]
Opinions about MA using social media	China	41782 MA-related posts were collected from social media data searched using a transfer learning technique	Regarding personal posts, 28.77% were negative, 22.91%, were neutral, and 48.32% showed positive feelings toward MA	[85]
Barriers, drivers, acceptability of, and perceptions towards PBM	Italy	Attitude–behavior–context model to analyze consumer behavior with respect to PBM	Key factors: knowledge, price, meat attachments, health and environmental concerns, and contextual aspects. Age, gender, and living state had no relation to acceptability. Taste was found to be the main barrier	[86]
Consumer preferences and the effects of brands with respect to FRM, CM, and PBMA	USA	Survey conducted according to the DCE method (>1800 participants). Four burger patties were evaluated using CM, PBAHP, PBM, and FRM	Vegetarian, younger, and educated people preferred CM and PBM. Price and brand names influence the buying intentions with respect to meat	[5]
Major barriers and motivators for shifting toward MA	Sweden	A stated preference survey: What price would make customers opt for an alternative burger instead of a regular one?	Less educated males and older subjects showed a stronger resistance, while young, environmentally, and health-conscious people were willing to accept PBM substitutes	[28]
Relevant factors in the acceptance of meat or PBMA products	Brazil	199 participants were subjected to eight distinct choice conditions (online survey)	PBMAs were accepted for easy-to-prepare (hamburgers) and basic foods. Products with functional benefits were preferred	[51]
Factors influencing PBM purchase decisions	Taiwan	234 females and 202 males answered the questionnaire on buying intentions with respect to PB burgers based on product information and environmental concerns	Consumers’ subjective norms and product knowledge have no relation to buying intentions, while perceived behavioral control and environmental concerns affect purchase intentions	[37]
The effect of marketing cues on consumers’ preferences for PBMA	Canada	149 undergraduate students participated in an MRP (15 min online session)	Product descriptors and packaging colors somehow affect the preferences and behavioral intentions of consumers	[7]
The market potential for PBMA in different contexts and settings	US	The survey (4894 responses) included four studies linked to consumer decisions made in restaurants and grocery stores	Sales of beef were not affected by introducing ingredient or nutritional details on PBM products. Little cross-price flexibility was noticed between PB patties and ground-beef patties	[35]
The market-driven transition of MA in the food markets	Finland	A dual dynamic of attachment and detachment was used to evaluate the market’s transition to MA	To attract customers, MA companies should fit their products to the present regime, cultural meaning, and values of meat in everyday food practices	[36]
Market expenditure data analysis for demand review for PBM vs. meat	40 states in the USA	Evaluation of recent 3-year trends for PBMA expenditure by retail-level Nielsen track scanner records (weekly)	Sales of PBMA are increasing significantly, although the market’s existing demand for PBM is still incomparable with that for meat	[54]
Consumption trends linked with PBAFs	UK	Repeated cross-sectional data from the NDNS (15,655 participants). Annual dietary intake and nutritional status detail collection	PBAF consumption increased from 6.7% in 2008–2011 to 13.1% in 2017–2019, proving that PBM is being accepted by UK consumers	[73]
Meat consumption and consumers’ shifts toward MA	Spain	Investigation about people’s attitudes and shifting trends and the availability of MA in food markets	MA is available in food stores across the whole country and acceptability is increasing. A decline in meat consumption is expected in future	[87]
Changes in theconsumption of beef, PBM, and IBM	Finland	Observations of dietary plans and shifts in 18–79-year-old people (N = 1000)	26% of participants planned to increase PBM consumption and 27% of respondents intended to reduce beef consumption in the future	[34]
Taste testing of beef, blended, pea protein, and PBM burgers	USA	Blind and informed (ingredient information) taste testing	Beef burgers were chosen over MA in informed testing, while in the blind test blended burgers were preferred. A pea protein burger was preferred over a PBM burger	[88]
Role of sensory attributes on the acceptability of MA	France	Four recipes prepared with various PBMA, and meat ingredients were evaluated by 160 people using a liking scale and CATA characterizations	Consumer liking was dependent on the products’ compositions, recipes, and some sensory attributes	[79]
The effect of product details on the consumption of beef, PB, and a hybrid burger	UK	Testing the burgers under blind, informed, and anticipated situations. Consumer responses were assessed in terms of liking, CATA, and willingness to buy	Consumers preferred blended burgers, considering overall acceptability, buying intentions, and individual comments	[80]
Effect of labels on the consumption of PBM and CM over traditional pork products	China	A food choice testing survey (3015 urban people) analyzing the efficacy of food labels (concerns related to health, animal safety, and the environment) and eating location	Food identity labels enhanced relative demand for AM products. Eating location did not affect meat choice. Older, female, and wealthier individuals preferred AM	[43]
Effect of labeling terminology on eating intentions with respect to PBM products	UK	The online study was conducted in two parts: 1. Do MA product labels (meat/vegetarian-related) appeal to people’s preferences? 2. The moderating role of gender on label preferences	1. Willingness to eat was higher when meat substitutes had meat-associated labels. 2. Labeling preferences respecting meat were independent of gender	[89]
Consumer insight into PB burger recipes	Denmark	PB burger recipes were analyzed for ingredients, length of recipes, clean labels, and ingredient familiarity using projective mapping	The number of ingredients is more important for consumer perception of clean-label status for PBM than the presence of chemical additives	[42]
Influence of cooking ability and FN on AM acceptance	Sweden	Consumers with differing cooking abilities and degrees of food neophobia assessed AM and minced-beef-based Bolognese sauce	Cooking ability and food neophobia were negatively related. Bolognese sauce samples were preferred regarding flavor and taste	[3]
Redesigning the restaurant menu to perceive PBMA as the default	Netherlands	Online and restaurant field study to increase the consumer selection of PBM over meat-based dishes	When a plant-based alternative is presented as the default, people choose PBMA more frequently than meat	[90]
Experience of meat-loving men with PBMA in a restaurant	Sydney	Qualitative in-depth interviews with 36 males who had eaten a PB burger at a vegan restaurant	Men affirmed the significance of women for the vegan restaurant visit, but they were unwilling to permanently add PBMA into their diets	[91]
Attitudes of millennial consumers with respect to TM vs. MA	Finland	A market research company was employed to collect questionnaire data	People who do not eat much meat or who want to reduce meat consumption have positive attitudes toward MA	[25]
Social and economic opportunities and difficulties with CM or PBM for rural producers	USA	37 experts (delegates of CM and PBM firms, non-profit/governmental organizations, researchers, beef/soy/pea industry representatives, and farmers) joined semi-organized interviews	Opportunities: Growing crops as ingredients for PBM, transitioning into new sectors. Threats: Farmers and livestock vendors who grow animal feed may experience income losses	[81]
Female (regular meat user) behavior with respect to MA containing rapeseed protein	Romania, Denmark, Iceland, Finland, and Germany	Online survey involving 1397 female contributors (main household decision makers)	The main ingredients of MA may boost or inhibit acceptance of PBM and lead to its being substituted for animal meat	[92]
Environmental and situational factors affecting IBM, CM, PBMA, or 3D-printed food consumption	Japan	Survey on Qualtrics involving 117 participants	Social companions and venues affect acceptance and choice of meat in different ways depending on the unfamiliarity or newness of the food type	[40]
Children’s behavior (8–10 years) towards PBM	Netherlands	Formal interviews with children (N = 34)	Food preferences and opinions of the children greatly affect the food choices of parents for the main meals	[93]

PBM = Plant-based meat, CM = Cultured meat, AMP = Alternative meat product, DCE = Discrete choice experiment, PBAFs = Plant-based alternative foods, CAWI = Computer-assisted web interviewing, AM = Alternative meat, PBMA = Plant-based meat alternative, NZ = New Zealand, DR = Dominican Republic, PBAHP = Plant-based animal-like heme protein, IBM = Insect-based meat, FRM = Farm-raised meat, PRF = Protein-rich food, NDNS = National Diet and Nutrition, RAG-CG = Respondi AG, Cologne, Germany, FN = Food neophobia, MRP = Marketing Research Practicum, CATA = Check-all-that-apply, MSH = Meat-substitute hamburger.

## Data Availability

The data presented in this article are available on request from the corresponding author.
